# Point-of-care prediction model of loop gain in patients with obstructive sleep apnea: development and validation

**DOI:** 10.1186/s12890-022-01950-y

**Published:** 2022-04-25

**Authors:** Christopher N. Schmickl, Jeremy E. Orr, Paul Kim, Brandon Nokes, Scott Sands, Sreeganesh Manoharan, Lana McGinnis, Gabriela Parra, Pamela DeYoung, Robert L. Owens, Atul Malhotra

**Affiliations:** 1 Division of Pulmonary, Critical Care and Sleep Medicine, University of California, San Diego (UCSD), San Diego, CA 92037 USA; 2 Division of Cardiology, University of California, San Diego (UCSD), San Diego, CA 92037 USA; 3 Division of Sleep and Circadian Disorders, Brigham and Women’s Hospital, Harvard Medical School, Boston, MA USA

**Keywords:** Clinical decision rules, Sleep apnea, obstructive, Respiration, Precision medicine

## Abstract

**Background:**

High loop gain (unstable ventilatory control) is an important—but difficult to measure—contributor to obstructive sleep apnea (OSA) pathogenesis, predicting OSA sequelae and/or treatment response. Our objective was to develop and validate a clinical prediction tool of loop gain.

**Methods:**

A retrospective cohort of consecutive adults with OSA (apnea–hypopnea index, AHI > 5/hour) based on in-laboratory polysomnography 01/2017–12/2018 was randomly split into a training and test-set (3:1-ratio). Using a customized algorithm (“reference standard”) loop gain was quantified from raw polysomnography signals on a continuous scale and additionally dichotomized (high > 0.7). Candidate predictors included general patient characteristics and routine polysomnography data. The model was developed (training-set) using linear regression with backward selection (tenfold cross-validated mean square errors); the predicted loop gain of the final linear regression model was used to predict loop gain class. More complex, alternative models including lasso regression or random forests were considered but did not meet pre-specified superiority-criteria. Final model performance was validated on the test-set.

**Results:**

The total cohort included 1055 patients (33% high loop gain). Based on the final model, higher AHI (beta = 0.0016; P < .001) and lower hypopnea-percentage (beta = −0.0019; P < .001) predicted higher loop gain values. The predicted loop gain showed moderate-to-high correlation with the reference loop gain (r = 0.48; 95% CI 0.38–0.57) and moderate discrimination of patients with high versus low loop gain (area under the curve = 0.73; 95% CI 0.67–0.80).

**Conclusion:**

To our knowledge this is the first prediction model of loop gain based on readily-available clinical data, which may facilitate retrospective analyses of existing datasets, better patient selection for clinical trials and eventually clinical practice.

**Supplementary Information:**

The online version contains supplementary material available at 10.1186/s12890-022-01950-y.

## Background

Approximately one billion people worldwide have obstructive sleep apnea (OSA), which is characterized by a repetitive collapse of the upper airway during sleep [1, 2], and associated with severe neurological (e.g., daytime sleepiness, traffic/work accidents) and cardiovascular (e.g., myocardial infarction, stroke) sequelae [2]. OSA is increasingly recognized as a mechanistically heterogeneous disease, explaining much of the variability in response to clinical and investigational therapies [3, 4].

Individuals with a predisposition to upper airway collapse can develop OSA via several different mechanisms (endotypes) including an unstable ventilatory control (high loop gain) which plays an important pathogenetic role in one third of OSA patients [5]. Loop gain is an engineering term used to describe an individual’s propensity for fluctuation in ventilation in response to a disturbance. An individual with high loop gain tends to have periodic drops in respiratory drive which result in reduced activation of upper airway dilators and thus can directly lead to repetitive respiratory events (i.e., OSA). Importantly, patients with the high loop gain endotype may also have a phenotype (clinical expression of disease) that includes increased risk of cardiovascular sequelae from OSA, and respond poorly to CPAP, hypoglossal nerve stimulation, or upper airway surgeries [6–10]. Conversely, high loop gain patients are more likely to benefit from loop gain-lowering interventions such as oxygen or acetazolamide [11–14].

Loop gain can be measured from flow signals obtained during overnight sleep studies using a customized algorithm programmed in MATLAB or Python [11, 15, 16]. However, this technique requires the availability of the raw data (including staging, arousal, and respiratory event annotations), some technical skills, and 20–40 min of computing time. To support efforts towards a personalized medicine approach for OSA our objective was to develop and validate a prediction model of loop gain, based on readily available clinical data allowing point-of-care assessments.

## Methods

We assembled a retrospective cohort of consecutive adults with OSA based on an apnea–hypopnea index (AHI, hypopnea-definition: peak flow reduction by ≥ 30% for ≥ 10 s associated with a ≥ 3% desaturation or cortical arousal) [17, 18] > 5events/hour on an in-laboratory polysomnography at UCSD between 01/2017 and 12/2018 (UCSD HRPP #182136XL; requirement for informed consent was waived in accordance with 45 CFR46.116(d)).

Using a customized MATLAB-algorithm (“reference standard”) loop gain was quantified from polysomnography data on a continuous scale (0 to infinity, dimensionless) [11]. Loop gain was additionally dichotomized (high vs. low) based on an established cut-off of 0.7 [11, 19].

Candidate predictors were chosen primarily based on prior knowledge [8, 10, 11, 13, 19–24]. Predictor data were abstracted from medical record notes/reports (available at the time of the polysomnography) without knowledge of the loop gain data:

- Demographics: age (years), sex (female/male), body mass index (kg/m^2^), race (White/Black/Asian/Other)
- Routine polysomnography results: AHI (/h), SpO_2_ nadir (%), SpO_2_ mean (%), percentage of hypopneas (0–100%), log-transformed AHI_REM/NREM_-ratio, total arousal index (/h)
- Miscellaneous: Hypertension (yes/no), heart failure (yes/no), atrial fibrillation (yes/no), prior stroke (yes/no); Supplemental oxygen use (yes/no); Ticagrelor use (yes/no); Family history of OSA (yes vs. no/unclear); current smoking or alcohol use (yes vs. no/unclear)

Recognizing the potential risks [25] of including race as a predictor, we considered it as a candidate because prior data [12, 19] suggested a physiological relationship with loop gain.

We excluded subjects with central sleep apnea (small subgroup in which predictors of loop gain may differ from those in OSA patients), missing loop gain (unable to retrieve raw data, or insufficient signal data for the algorithm to estimate loop gain) or missing predictor data (missingness was minimal thus we chose a complete-case analysis over imputation methods).

### Analysis

The dataset was randomly split into a training versus test-set (3:1-ratio). All feature selection and model comparison procedures were performed on the training-set. The performance of the final model was assessed on the test-set. All analyses were performed in R (3.6.1); key packages included leaps (backward selection), glmnet (lasso), randomForest and pROC. The analytical approach and reporting followed recently published expert-recommendations, guidelines and the TRIPOD-checklist [25–27].

### Objective 1: to predict loop gain as a continuous outcome

#### Training-set

For model building we primarily focused on linear regression given its widespread use and high interpretability. Features were selected in a three-step process: i) backward selection to create p (p = number of potential predictors) candidate models (with 1, 2,…, p − 1, p predictors); ii) for each of the p candidate models the test error (mean square error, MSE) was directly estimated using tenfold cross validation (CV); iii) to balance predictive power with parsimony and avoid overfitting, the final linear regression model was selected based on the lowest CV-MSE applying the *one-standard error rule* [26]. The need for polynomials or interaction terms was assessed similarly.

Using the final linear regression model as a benchmark, we assessed if more complex modelling approaches would yield superior predictive accuracy based on the following criterion:

$${\text{Estimated\,test\,MSE}}_{{{\text{Alternative.Model}}}} < \left[ {{\text{CV-MSE}}{-}{1}\,{\text{standard\,error}}} \right]_{{{\text{Final.Linear.Regression.Model}}}}$$

The first alternative model which we considered, was a linear regression model with feature selection based on lasso (selecting the tuning parameter lambda based on the lowest CV-MSE applying the *one-standard error rule*). The second alternative model was based on a random regression forest (N_Trees_ = 500, considering p/3 predictors at each split). The estimated test errors for these two alternative models were based on the tenfold CV-MSE and the “out-of-bag” MSE, respectively.

#### Test-set

We assessed the performance of the final prediction model based on the root mean square error (RMSE), Pearson r, and visual inspection of a regression of the predicted on the reference loop gain values. This approach was chosen over Bland–Altman analyses, because the latter implicitly assumes that the “true loop gain” lies between the estimates from both methods [28], whereas our goal was to provide a precise and accurate prediction of the reference standard.

### Objective 2: to predict loop gain as a binary outcome (high vs. low)

#### Training-set

For simplicity, we primarily used the predicted loop gain value of the final linear regression model to predict loop gain class (i.e., using different cut-offs of the predicted loop gain value to classify subjects as having high vs. low loop gain). We estimated the discriminative value of this approach using tenfold CV to estimate directly the test area under the receiver operating curve (AUC) and its standard error. Using the linear regression model as a benchmark, we then assessed if more complex modelling approaches would yield superior predictive performance based on the following criterion:

$${\text{Estimated\,test\,AUC}}_{{{\text{Alternative.Model}}}} > \left[ {{\text{CV-AUC}} + {1}\,{\text{standard\,error}}} \right]_{{{\text{Final.Linear.Regression.Model}}}}$$

The alternative models were based on logistic regression using backward selection or lasso for feature selection, and a random forest classifier (N_Trees_ = 500, considering p^0.5^ predictors at each split).

#### Test-set

The performance of the final classifier model was primarily quantified by the AUC. Additionally, sensitivity, specificity, and positive/negative predictive values (+ bootstrapped 95% confidence intervals) were calculated for a range of potential threshold values.

### Sample size

There are no generally accepted approaches for sample size calculations for prediction studies, thus we used all available data to maximize power and generalizability [27]. Of note, our training-set exceeded 10 “events” (i.e., “high” loop gain) per candidate predictor, which is often used as a rule of thumb to assess adequacy of sample size for the development of classifier models [27].

## Results

**Fig. 1:**
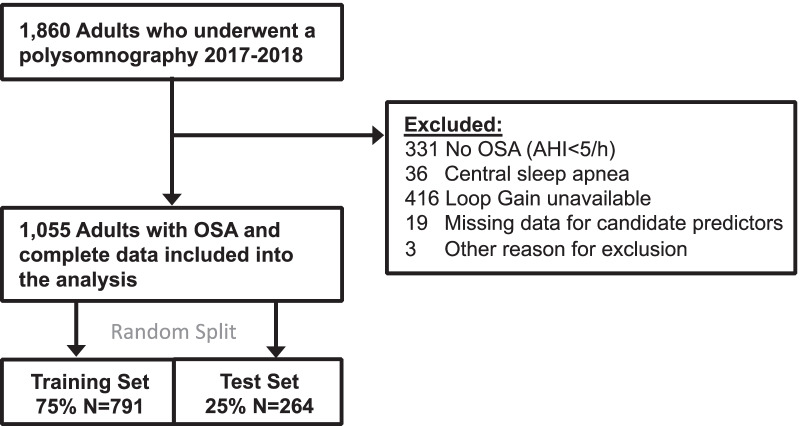
Study flowchart

We included 1055 subjects into the analysis (Fig. 1). Table 1 provides details of the cohort, which was notable for a broad age range (mean 55 years [standard deviation 15]), including 44% women and > 40% non-Whites. One third of subjects had high loop gain based on the signal analysis (reference standard). Compared with patients who had low loop gain, high loop gain patients were older, more likely male, had more severe OSA, and shorter respiratory events. Of note, almost all subjects on ticagrelor had high loop gain, but the total number of subjects taking this medication was small (N = 6).

**Table 1:** General characteristics of included subjects

Characteristic	Based on loop gain > 0.7			
	Overall N = 1055^1^	Low N = 711 (67%)^1^	High N = 344 (33%)^1^	p-value^2^
Loop gain (dimensionless)	0.65 (0.21)	0.54 (0.11)	0.88 (0.16)	< 0.001
*Demographics, social and family history*				
Age (y)	55 (15)	54 (15)	58 (14)	< 0.001
Female sex	465 (44%)	332 (47%)	133 (39%)	0.014
BMI (kg/m^2^)	33 (9)	33 (9)	33 (8)	0.8
Race				0.5
White	617 (58%)	410 (58%)	207 (60%)	
Black	76 (7.2%)	48 (6.8%)	28 (8.1%)	
Asian	88 (8.3%)	64 (9.0%)	24 (7.0%)	
Other/unclear	274 (26%)	189 (27%)	85 (25%)	
Current smoking	69 (6.5%)	47 (6.6%)	22 (6.4%)	> 0.9
Current alcohol use	456 (43%)	304 (43%)	152 (44%)	0.7
OSA family history	104 (9.9%)	68 (9.6%)	36 (10%)	0.7
*Co-morbidities and medications*				
Hypertension	488 (46%)	315 (44%)	173 (50%)	0.075
Heart failure	65 (6.2%)	41 (5.8%)	24 (7.0%)	0.5
Arial fibrillation	74 (7.0%)	48 (6.8%)	26 (7.6%)	0.6
Prior stroke	32 (3.0%)	23 (3.2%)	9 (2.6%)	0.7
Oxygen use	45 (4.3%)	29 (4.1%)	16 (4.7%)	0.7
Ticagrelor	6 (0.6%)	1 (0.1%)	5 (1.5%)	0.016
*Data from the polysomnography-report*				
AHI (/h)	35 (29)	29 (26)	46 (32)	< 0.001
SpO_2_ nadir (%)	80 (9)	81 (9)	79 (11)	0.001
SpO_2_ mean (%)	93.50 (2.53)	93.7 (2.41)	93.1 (2.75)	0.002
Percent hypopneas (%)	70 (27)	75 (24)	59 (29)	< 0.001
Mean event duration (s)	24 (6)	24 (6)	22 (6)	< 0.001
Total arousal index (/h)	30 (24)	26 (20)	37 (28)	< 0.001
Log-AHI_REM/NREM_-ratio^3^	0.20 (1.18)	0.29 (1.25)	0.01 (1.00)	< 0.001

All characteristics (other than loop gain) were considered candidate predictors

^1^Mean (SD); n (%)

^2^Welch Two Sample t-test; Fisher's exact test

^3^If REM sleep was absent, then AHI_REM_ was assumed to equal AHI_NREM_; if the log-transformed REM/NREM-AHI-ratio was—infinity [i.e., AHI_REM_ = 0], then—infinity was imputed as the smallest observed value of 3.91

### Predicting continuous loop gain

Based on backward selection using tenfold-CV MSE the optimal linear regression model contained only two predictors, the AHI and the percentage of hypopneas (E-Appendix 1).

The lasso regression model selected the same two predictors (E-Appendix 2), which were also identified as the two most important predictors by the random forest model (E-Appendix 3). Neither the lasso regression model nor the random forest model met our criteria for superiority compared to the less complex standard linear regression model (Additional file 1: Fig. S1) which was thus selected as the final prediction model (Table 2). Based on this model, a higher AHI and a lower percentage of hypopneas predicted a higher loop gain.

**Table 2:** Final linear regression model

	Estimate	Standard Error	t value	p-value
(Intercept)	0.72290	0.02531	28.56	< 0.001
Apnea Hypopnea Index (/h)	0.00159	0.00026	6.12	< 0.001
Percentage of Hypopneas (%)	− 0.00186	0.00027	− 6.76	< 0.001

**Fig. 2:**
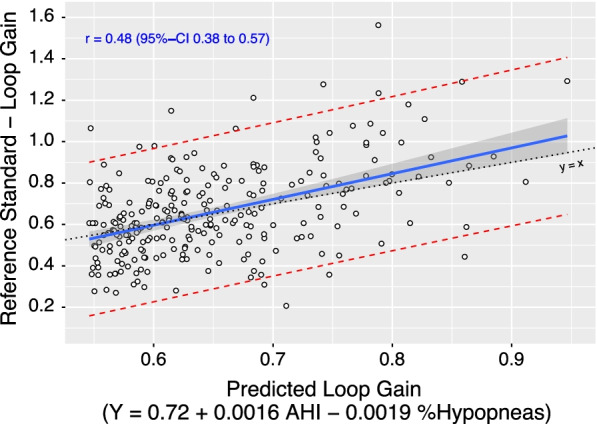
Predicted loop gain (linear regression model) versus the reference loop gain (raw signal analysis) based on the test set data. The blue solid line shows the mean reference loop gain for a predicted loop gain and its 95%-confidence interval, which includes the dotted line of identity (i.e., no bias). The red dashed lines show the 95%-prediction interval

On the test set, the RMSE (“average prediction error”) was 0.19 and there was a moderate-to-high [29] correlation based on Pearson’s r = 0.48 (95% CI 0.38–0.57) between the predicted and the reference-loop gain. Figure 2 visualizes the spread of actual reference loop gains for a given predicted loop gain, demonstrating that there was no substantial bias across the range of predicted loop gain values (i.e., 95% interval of the least-squares mean of the reference loop gain includes the line of identity throughout).

### Predicting categorical loop gain (high vs. low)

The logistic regression models based on backward selection (E-Appendix 4) and lasso (E-Appendix 5) selected 3 (age, percent-hypopneas, mean event duration) and 5 predictors (AHI, percent-hypopneas, age, ticagrelor, mean event duration) for the optimal model, respectively. Despite the larger number of predictors, the alternative models which further included a random forest classifier model (E-Appendix 6) did not meet criteria for superiority relative to the simple 2-predictor linear regression model (Additional file 1: Fig. S2) which was thus also selected as the final classifier model (Table 2).

**Fig. 3:**
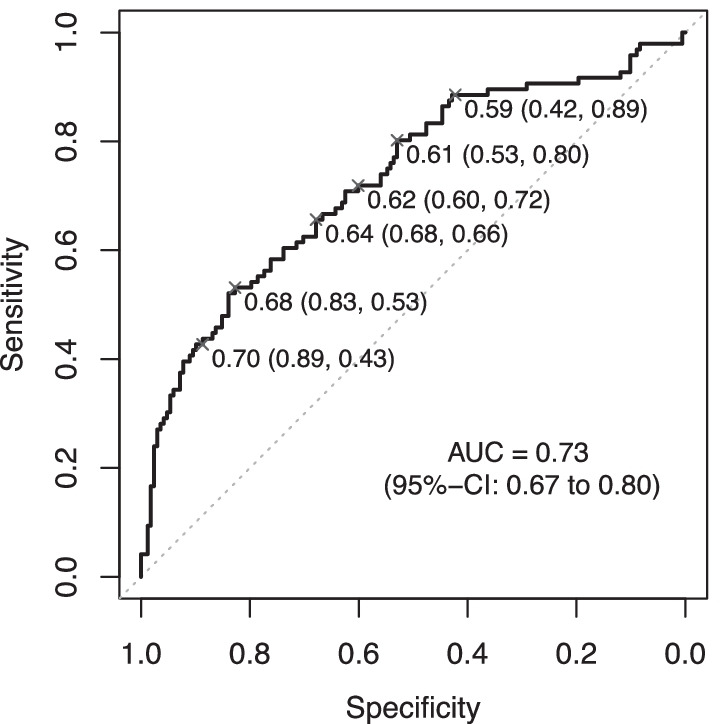
AUC of the final classifier model (i.e., the linear regression model shown in Table 2). Select thresholds are marked by an “x” followed by the “threshold value (specificity, sensitivity)”. For more details about various thresholds see Table 3

On the test set, the linear model demonstrated moderate discrimination (AUC = 0.73; 95% CI 0.67–0.80; Fig. 3). Sensitivity and specificity, as well as positive/negative predictive values for various cut-off values are shown in Table 3. In patients with a predicted “low” loop gain (cut-off_Youden’s Index_ = 0.682), the mean reference loop gain was 0.60 (95% CI 0.58–0.63) compared with 0.79 (95% CI 0.73–0.85) in those predicted to have “high” loop gain, suggesting clinically meaningful separation by the model.

**Table 3:** Performance characteristics for varying thresholds

Threshold	Sensitivity	(95% CI)	Specificity	(95% CI)	PPV	(95% CI)	NPV	(95% CI)
0.55	0.98	(0.95–1)	0.02	(0.01–0.05)	0.36	(0.36–0.37)	0.67	(0.25–1)
0.56	0.94	(0.89–0.98)	0.10	(0.06–0.15)	0.37	(0.36–0.39)	0.75	(0.55–0.9)
0.57	0.92	(0.85–0.97)	0.20	(0.14–0.26)	0.39	(0.37–0.42)	0.81	(0.69–0.91)
0.58	0.90	(0.83–0.95)	0.29	(0.22–0.36)	0.42	(0.39–0.45)	0.83	(0.74–0.92)
0.59	0.89	(0.82–0.95)	0.38	(0.31–0.46)	0.45	(0.42–0.48)	0.85	(0.78–0.92)
0.60	0.84	(0.77–0.92)	0.45	(0.38–0.52)	0.47	(0.43–0.51)	0.84	(0.76–0.9)
0.61	0.81	(0.73–0.89)	0.51	(0.42–0.58)	0.48	(0.44–0.53)	0.82	(0.76–0.89)
0.62	0.73	(0.65–0.81)	0.56	(0.48–0.63)	0.49	(0.43–0.54)	0.78	(0.73–0.84)
0.63	0.67	(0.57–0.76)	0.65	(0.57–0.72)	0.52	(0.46–0.59)	0.78	(0.72–0.83)
0.64	0.65	(0.55–0.74)	0.68	(0.6–0.75)	0.54	(0.47–0.6)	0.77	(0.72–0.82)
0.65	0.60	(0.51–0.71)	0.74	(0.67–0.8)	0.57	(0.5–0.65)	0.77	(0.72–0.82)
0.66	0.56	(0.46–0.66)	0.77	(0.7–0.83)	0.58	(0.51–0.66)	0.76	(0.71–0.8)
0.67	0.54	(0.45–0.65)	0.79	(0.72–0.85)	0.59	(0.51–0.68)	0.75	(0.71–0.8)
0.68	0.53	(0.44–0.64)	0.83	(0.76–0.88)	0.64	(0.55–0.73)	0.76	(0.71–0.8)
0.69	0.46	(0.35–0.56)	0.85	(0.79–0.9)	0.64	(0.54–0.74)	0.73	(0.7–0.77)
0.70	0.43	(0.33–0.53)	0.89	(0.83–0.93)	0.68	(0.58–0.79)	0.73	(0.7–0.77)
0.71	0.42	(0.32–0.52)	0.90	(0.85–0.94)	0.70	(0.59–0.81)	0.73	(0.7–0.76)
0.72	0.41	(0.31–0.51)	0.91	(0.86–0.95)	0.72	(0.61–0.84)	0.73	(0.7–0.76)
0.73	0.39	(0.29–0.49)	0.92	(0.88–0.96)	0.74	(0.62–0.85)	0.72	(0.69–0.76)
0.74	0.33	(0.24–0.43)	0.94	(0.9–0.97)	0.76	(0.64–0.88)	0.71	(0.68–0.74)
0.75	0.29	(0.2–0.39)	0.95	(0.92–0.98)	0.78	(0.65–0.9)	0.70	(0.68–0.73)
0.76	0.27	(0.19–0.36)	0.97	(0.94–0.99)	0.84	(0.7–0.96)	0.70	(0.67–0.73)
0.77	0.24	(0.16–0.32)	0.98	(0.95–0.99)	0.86	(0.71–0.97)	0.69	(0.67–0.72)
0.78	0.21	(0.12–0.29)	0.98	(0.95–0.99)	0.84	(0.68–0.97)	0.68	(0.66–0.71)
0.79	0.17	(0.09–0.24)	0.98	(0.95–0.99)	0.81	(0.61–0.96)	0.67	(0.65–0.69)
0.80	0.12	(0.06–0.2)	0.98	(0.96–1)	0.80	(0.57–1)	0.66	(0.65–0.68)

95% confidence intervals (CI) were estimated based on a bootstrap procedure (N_samples_ = 2000)

*PPV* positive predictive value, *NPV* negative predictive value

Lastly, we explored if there may be better models to predict loop gain in the subgroup of patients with moderate/severe OSA (i.e., AHI > 15/h). However, these exploratory analyses suggest that the final linear model developed from the full cohort is also the optimal model for—and performs well in—the subgroup of patients with moderate/severe OSA (Additional file 1: E-Appendix 7).

## Discussion

Using state-of-the art methodology we developed and internally validated a point-of-care model to predict (high) loop gain in patients with OSA, which showed moderate predictive performance. We note several important findings:

First, while we considered a broad range of candidate features—somewhat surprisingly, but similar as in other recent studies aiming to predict OSA traits [24, 30]—we found that most of the predictive information is contained in just two polysomnographic variables, the AHI and the percentage of hypopneas. Importantly, a high percentage of hypopneas (i.e., hypopneas > apneas) is often considered a marker for OSA with only a mild-moderate anatomical collapsibility in which non-anatomical traits such as a low arousal threshold play a greater pathophysiological role [9, 30, 31]. Thus, interventional studies targeting non-anatomical traits are increasingly considering a high percentage of hypopneas as an inclusion criterion (e.g., NCT04639193). However, our results suggest that for studies targeting the non-anatomical trait loop gain (e.g., via acetazolamide/oxygen) this approach may exclude the very patients expected to be most responsive (i.e., patients with high loop gain) [11–13] Similarly, our model further suggests that using the AHI alone may be a poor selection criterion for such studies. Instead, we propose consideration of our prediction score applying a threshold that provides the desired sensitivity/specificity (Table 3).

Second, while the performance of our model may be adequate for certain research/clinical scenarios, there was much variability in reference-loop gain values which was not explained by routinely available data. This finding suggests that the raw flow signals encode some important physiological information not readily captured by routine clinical data, thus emphasizing the importance of continued efforts to increase scalability and portability of such analyses [11, 15, 16, 32].

Third, some may consider loop gain estimates based on the pressure drop technique during a specialized research study as a more appropriate reference standard to develop a prediction model [24, 33]. However, due to the labor-intensive nature of this technique, datasets are typically small posing a power challenge for the development of prediction models [24]. More importantly, one may argue that there is no real “gold standard” to measure loop gain, but the primary purpose of loop gain estimation is typically to match OSA patients with personalized treatments. As such, the ideal benchmark to assess the (criterion) validity for any loop gain model is the prediction of clinically important outcomes (e.g., response to surgeries or drug therapies), which is increasingly well documented for the reference standard that we used [6, 7, 9, 11, 14, 34] Interestingly, a “self-similarity” metric has been recently shown to predict residual central events (a clinical consequence of high loop gain), but this technique requires advanced signal analyses, and has not yet been externally validated or shown to predict other loop gain related outcomes [35].

Fourth, using a tree-based machine learning approach and loop gain from the pressure drop technique Dutta et al. [24] were able to predict the traits “anatomical collapsibility” and “arousal threshold” with moderate accuracy from clinical data on highly selected research subjects with and without OSA; but their predictive accuracy of loop gain was similar to chance. Using a different “reference standard” (see above) we were able to assemble a development cohort of general sleep clinic patients that was > 15 times larger thus providing greater power and generalizability. Thus, to our knowledge we present here the first applicable clinical prediction model of loop gain. We anticipate that his model may serve a similar role as the clinical prediction score for the arousal threshold by *Edwards *et al*.* which has enabled several retrospective analyses of existing OSA cohorts thus generating important clinical insights [30, 36–38]. We further note, that in some settings it may be desirable to identify patients who have both a mild anatomical collapsibility and high loop gain, which could well be achieved by applying the model from *Dutta *et al. together with ours [24].

Strengths of our study include the large cohort of patients which are reflective of the target population, and the consideration of a wide range of advanced statistical techniques including a non-linear random forest model to ensure that simple linear modelling did not result in an excessive loss of predictive performance. Further, the similar results across the various candidate models (e.g., feature selection) suggests robustness of our results.

As discussed above, a major limitation is the lack of a clear gold standard for loop gain and the lack of external validation. Other limitations include the potential dependence of our model performance on the scoring definition for hypopneas that we used [17, 18], and that our findings may not generalize to patients whose OSA was diagnosed based on a home sleep apnea test (HSAT) as such data/patients were not included in the present study. However, we note that many HSATs include information on a surrogate AHI as well as the percentage of hypopnea, thus it may be possible to “calibrate” the estimates from our model for this setting. Importantly, we have previously demonstrated that loop gain can be estimated from the HSAT flow signals in a similar manner as from polysomnography, which could thus serve as the reference standard for such a calibration attempt in the future [15].

## Conclusions

Together, the AHI and the percentage of hypopneas allow clinical prediction of loop gain with moderate accuracy. This prediction model may facilitate better patient selection for clinical trials: patients predicted to have “high” loop could be preferentially selected for studies of loop gain lowering interventions (i.e., acetazolamide, oxygen), but be excluded from interventions that target other traits such as anatomy (i.e., upper airway surgery, oral appliance, hypoglossal nerve stimulation). Importantly, the provided information about sensitivities/specificity for various threshold values (Table 3) allows a highly flexible implementation according to individual contexts and needs.

## Supplementary Information

**Additional file 1**. Online Supplement.

## Data Availability

The data that support the findings of this study are available from the corresponding author upon reasonable request and approval of the UCSD Human Research Protections Program.

## Abbreviations

AHI: Apnea-hypopnea index
AUC: Area under the receiver operating characteristic curve
CV: Cross-validation
(R)MSE: (Root) mean square error
OSA: Obstructive sleep apnea
