# Supplementary material for: Point-of-care prediction model of loop gain in patients with obstructive sleep apnea: development and validation
Source: BMC Pulm Med. 2022 Apr 25;22:158. doi: 10.1186/s12890-022-01950-y (PMC9036750; doi:10.1186/s12890-022-01950-y)

**ONLINE SUPPLEMENT**

**Point-of-Care Prediction of Loop Gain in Patients with Obstructive Sleep Apnea: Development and Validation**

**Table of Content:**

[E-Appendix 1. Details of Backward Model Selection for the Linear Regression Model. 2](#_Toc99540290)

[E-Appendix 2. Details of the Linear Regression Model Based on Lasso. 4](#_Toc99540291)

[E-Appendix 3. Relative Importance of Predictors for Continuous Loop Gain based on the Random Regression Forest Model. 5](#_Toc99540292)

[E-Appendix 4. Details of the Backward Selection for the Logistic Regression Model. 6](#_Toc99540293)

[E-Appendix 5. Details of the Logistic Regression Model Based on Lasso. 8](#_Toc99540294)

[E-Appendix 6. Relative Importance of Predictors for Categorical Loop Gain (High vs Low) based on the Random Forest Classifier Model. 9](#_Toc99540295)

[E-Appendix 7. Model Development and Validation In the Subgroup of Patients with Moderate/Severe OSA 10](#_Toc99540296)

[E-Figure 1. Comparison of Prediction Models of Continuous Loop Gain. 13](#_Toc99540297)

[E-Figure 2. Comparison of Prediction Models of Categorical Loop Gain (High vs Low). The estimated test AUC for the linear regression model based on 10-fold cross-validation was 0.674. 14](#_Toc99540298)

# E-Appendix 1. Details of Backward Model Selection for the Linear Regression Model.

The model with 7 predictors had the lowest estimated test error (mean square error, MSE) based on 10-fold cross validation (red dot; red error bar = 1 standard error). However, the one standard error rule suggested similar performance for the 2-predictor model (green dot):


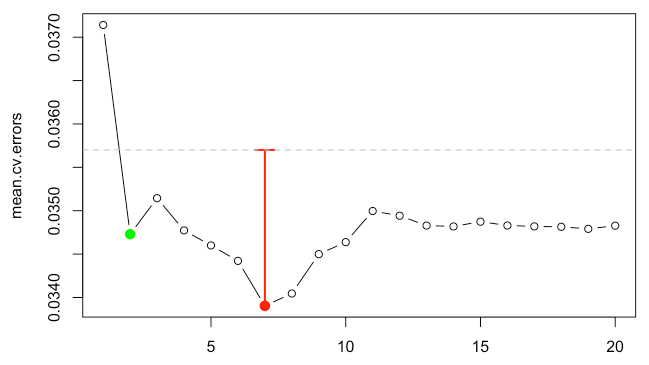


The table below shows which predictors were included in the 20 candidate models (“*”). Predictors which were included in more models can be considered of relative greater importance. The number on the left indicates the number of predictors included in a given model.

Age Sex Bmi RaceBlack RaceAsian RaceOther Smoking Alcohol FamilyHistory

1 ( 1 ) " " " " " " " " " " " " “ " " " " "

2 ( 1 ) " " " " " " " " " " " " " " " " " "

3 ( 1 ) " " " " " " " " " " " " " " " " " "

4 ( 1 ) " " " " "*" " " " " " " " " " " " "

5 ( 1 ) " " " " "*" " " "*" " " " " " " " "

6 ( 1 ) "*" " " "*" " " "*" " " " " " " " "

7 ( 1 ) "*" " " "*" " " "*" " " " " " " "*"

8 ( 1 ) "*" "*" "*" " " "*" " " " " " " "*"

9 ( 1 ) "*" "*" "*" " " "*" " " " " " " "*"

10 ( 1 ) "*" "*" "*" " " "*" " " " " " " "*"

11 ( 1 ) "*" "*" "*" " " "*" " " " " " " "*"

12 ( 1 ) "*" "*" "*" " " "*" " " " " " " "*"

13 ( 1 ) "*" "*" "*" " " "*" " " " " " " "*"

14 ( 1 ) "*" "*" "*" " " "*" " " " " " " "*"

15 ( 1 ) "*" "*" "*" " " "*" " " " " " " "*"

16 ( 1 ) "*" "*" "*" " " "*" "*" " " " " "*"

17 ( 1 ) "*" "*" "*" " " "*" "*" " " " " "*"

18 ( 1 ) "*" "*" "*" "*" "*" "*" " " " " "*"

19 ( 1 ) "*" "*" "*" "*" "*" "*" " " " " "*"

20 ( 1 ) "*" "*" "*" "*" "*" "*" "*" " " "*"

HTN CHF Afib Stroke OxygenUse Ticagrelor Ahi Spo2_nadir Spo2_mean %Hypopneas

1 ( 1 ) " " " " " " " " " " " " " " " " " " "*"

2 ( 1 ) " " " " " " " " " " " " "*" " " " " "*"

3 ( 1 ) " " " " " " " " " " " " "*" " " " " "*"

4 ( 1 ) " " " " " " " " " " " " "*" " " " " "*"

5 ( 1 ) " " " " " " " " " " " " "*" " " " " "*"

6 ( 1 ) " " " " " " " " " " " " "*" " " " " "*"

7 ( 1 ) " " " " " " " " " " " " "*" " " " " "*"

8 ( 1 ) " " " " " " " " " " " " "*" " " " " "*"

9 ( 1 ) "*" " " " " " " " " " " "*" " " " " "*"

10 ( 1 ) "*" " " " " " " " " "*" "*" " " " " "*"

11 ( 1 ) "*" " " " " " " " " "*" "*" " " " " "*"

12 ( 1 ) "*" " " " " " " " " "*" "*" " " "*" "*"

13 ( 1 ) "*" " " " " " " " " "*" "*" "*" "*" "*"

14 ( 1 ) "*" " " " " "*" " " "*" "*" "*" "*" "*"

15 ( 1 ) "*" " " "*" "*" " " "*" "*" "*" "*" "*"

16 ( 1 ) "*" " " "*" "*" " " "*" "*" "*" "*" "*"

17 ( 1 ) "*" " " "*" "*" " " "*" "*" "*" "*" "*"

18 ( 1 ) "*" " " "*" "*" " " "*" "*" "*" "*" "*"

19 ( 1 ) "*" " " "*" "*" "*" "*" "*" "*" "*" "*"

20 ( 1 ) "*" " " "*" "*" "*" "*" "*" "*" "*" "*"

Mean_event_duration Arous_Ind Log-AHI_rem/nrem

1 ( 1 ) " " " " " "

2 ( 1 ) " " " " " "

3 ( 1 ) "*" " " " "

4 ( 1 ) "*" " " " "

5 ( 1 ) "*" " " " "

6 ( 1 ) "*" " " " "

7 ( 1 ) "*" " " " "

8 ( 1 ) "*" " " " "

9 ( 1 ) "*" " " " "

10 ( 1 ) "*" " " " "

11 ( 1 ) "*" "*" " "

12 ( 1 ) "*" "*" " "

13 ( 1 ) "*" "*" " "

14 ( 1 ) "*" "*" " "

15 ( 1 ) "*" "*" " "

16 ( 1 ) "*" "*" " "

17 ( 1 ) "*" "*" "*"

18 ( 1 ) "*" "*" "*"

19 ( 1 ) "*" "*" "*"

20 ( 1 ) "*" "*" "*"

# E-Appendix 2. Details of the Linear Regression Model Based on Lasso.

The estimated test error based on 10-fold cross-validation (MSE, y axis) and the number of included predictors (top row) as a function of the tuning parameter lambda (x axis):


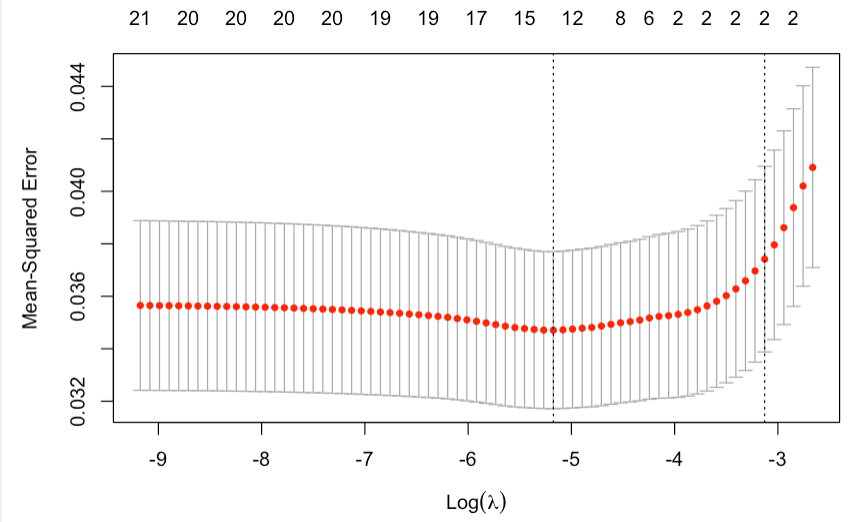


The Lambda of 0.0057 (log-lambda = -5.2) had the lowest MSE based on a 13-predictor model. However, the one standard error rule suggested similar performance for a much less complex 2-predictor model based on a lambda of 0.0438 (log-lambda = -3.1) which served as the comparator model for the almost identical final linear regression model. The table below shows the details of the lasso regression model:

|  | **Estimate** |
| --- | --- |
| (Intercept) | 0.6807 |
| Apnea Hypopnea Index (/h) | 0.000518 |
| Percentage of Hypopneas (%) | -0.000724 |

# E-Appendix 3. Relative Importance of Predictors for Continuous Loop Gain based on the Random Regression Forest Model.


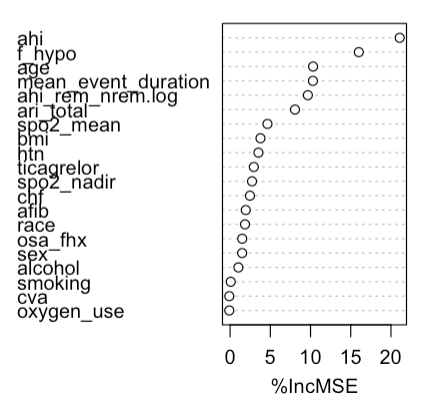


# E-Appendix 4. Details of the Backward Selection for the Logistic Regression Model.

The model with 10 predictors had the highest estimated test AUC (area under the receiver operating curve, AUC) based on 10-fold cross validation (red dot; red error bar = 1 standard error). However, the one standard error rule suggested similar performance for the 3-predictor model (green dot):


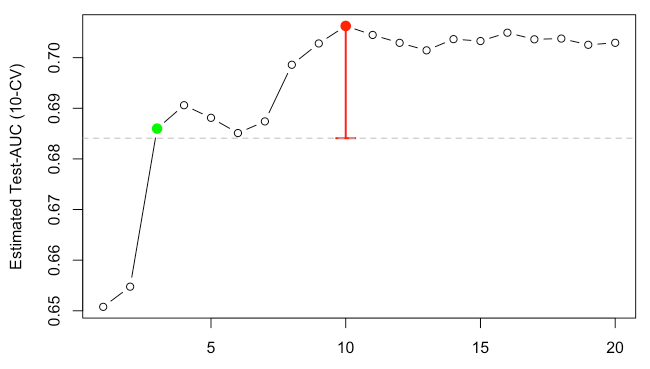


The table below shows which predictors were included in the 20 candidate models (“*”). Predictors which were included in more models can be considered of relative greater importance (note the very similar “importance” of predictors as for the linear regression model). The number on the left indicates the number of predictors included in a given model.

Selection Algorithm: backward

Age Sex Bmi RaceBlack RaceAsian RaceOther Smoking Alcohol FamilyHistory

1 ( 1 ) " " " " " " " " " " " " " " " " " "

2 ( 1 ) "*" " " " " " " " " " " " " " " " "

3 ( 1 ) "*" " " " " " " " " " " " " " " " "

4 ( 1 ) "*" "*" " " " " " " " " " " " " " "

5 ( 1 ) "*" "*" " " " " " " " " " " " " " "

6 ( 1 ) "*" "*" " " " " " " " " " " " " " "

7 ( 1 ) "*" "*" " " " " "*" " " " " " " " "

8 ( 1 ) "*" "*" "*" " " "*" " " " " " " " "

9 ( 1 ) "*" "*" "*" " " "*" " " " " " " "*"

10 ( 1 ) "*" "*" "*" " " "*" " " " " " " "*"

11 ( 1 ) "*" "*" "*" " " "*" " " " " " " "*"

12 ( 1 ) "*" "*" "*" " " "*" " " " " " " "*"

13 ( 1 ) "*" "*" "*" " " "*" " " " " " " "*"

14 ( 1 ) "*" "*" "*" " " "*" " " " " " " "*"

15 ( 1 ) "*" "*" "*" " " "*" " " "*" " " "*"

16 ( 1 ) "*" "*" "*" " " "*" " " "*" " " "*"

17 ( 1 ) "*" "*" "*" " " "*" " " "*" " " "*"

18 ( 1 ) "*" "*" "*" " " "*" "*" "*" " " "*"

19 ( 1 ) "*" "*" "*" " " "*" "*" "*" "*" "*"

20 ( 1 ) "*" "*" "*" " " "*" "*" "*" "*" "*"

HTN CHF Afib Stroke OxygenUse Ticagrelor Ahi Spo2_nadir Spo2_mean %Hypopneas

1 ( 1 ) " " " " " " " " " " " " " " " " " " "*"

2 ( 1 ) " " " " " " " " " " " " " " " " " " "*"

3 ( 1 ) " " " " " " " " " " " " " " " " " " "*"

4 ( 1 ) " " " " " " " " " " " " " " " " " " "*"

5 ( 1 ) " " " " " " " " " " " " "*" " " " " "*"

6 ( 1 ) " " " " " " " " " " "*" "*" " " " " "*"

7 ( 1 ) " " " " " " " " " " "*" "*" " " " " "*"

8 ( 1 ) " " " " " " " " " " "*" "*" " " " " "*"

9 ( 1 ) " " " " " " " " " " "*" "*" " " " " "*"

10 ( 1 ) " " " " " " " " " " "*" "*" " " " " "*"

11 ( 1 ) " " " " " " "*" " " "*" "*" " " " " "*"

12 ( 1 ) " " " " " " "*" " " "*" "*" " " "*" "*"

13 ( 1 ) " " " " " " "*" "*" "*" "*" " " "*" "*"

14 ( 1 ) " " " " " " "*" "*" "*" "*" " " "*" "*"

15 ( 1 ) " " " " " " "*" "*" "*" "*" " " "*" "*"

16 ( 1 ) "*" " " " " "*" "*" "*" "*" " " "*" "*"

17 ( 1 ) "*" " " "*" "*" "*" "*" "*" " " "*" "*"

18 ( 1 ) "*" " " "*" "*" "*" "*" "*" " " "*" "*"

19 ( 1 ) "*" " " "*" "*" "*" "*" "*" " " "*" "*"

20 ( 1 ) "*" "*" "*" "*" "*" "*" "*" " " "*" "*"

Mean_event_duration Arous_Ind Log-AHI_rem/nrem

1 ( 1 ) " " " " " "

2 ( 1 ) " " " " " "

3 ( 1 ) "*" " " " "

4 ( 1 ) "*" " " " "

5 ( 1 ) "*" " " " "

6 ( 1 ) "*" " " " "

7 ( 1 ) "*" " " " "

8 ( 1 ) "*" " " " "

9 ( 1 ) "*" " " " "

10 ( 1 ) "*" " " "*"

11 ( 1 ) "*" " " "*"

12 ( 1 ) "*" " " "*"

13 ( 1 ) "*" " " "*"

14 ( 1 ) "*" "*" "*"

15 ( 1 ) "*" "*" "*"

16 ( 1 ) "*" "*" "*"

17 ( 1 ) "*" "*" "*"

18 ( 1 ) "*" "*" "*"

19 ( 1 ) "*" "*" "*"

20 ( 1 ) "*" "*" "*"

# E-Appendix 5. Details of the Logistic Regression Model Based on Lasso.

The estimated test AUC based on 10-fold cross-validation (y axis) and the number of included predictors (top row) as a function of the tuning parameter lambda (x axis):


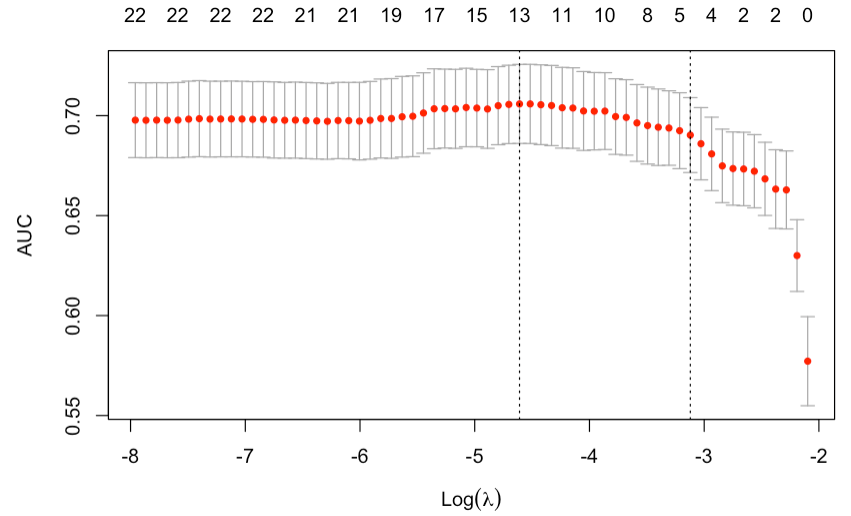


The Lambda of 0.00996 (log-lambda = -4.6) had the highest AUC based on a 13-predictor model. However, the one standard error rule suggested similar performance for a much less complex 5-predictor model based on a lambda of 0.04411 (log-lambda = -3.1) which served as the comparator model for the linear regression model. The table below shows the details of the lasso regression model:

|  | **Estimate** |
| --- | --- |
| (Intercept) | -0.39335 |
| Apnea Hypopnea Index (/h) | 0.00530 |
| Percentage of Hypopneas (%) | -0.01006 |
| Age | 0.00584 |
| Ticagrelor | 0.35829 |
| Mean Event Duration | -0.00940 |

# E-Appendix 6. Relative Importance of Predictors for Categorical Loop Gain (High vs Low) based on the Random Forest Classifier Model.


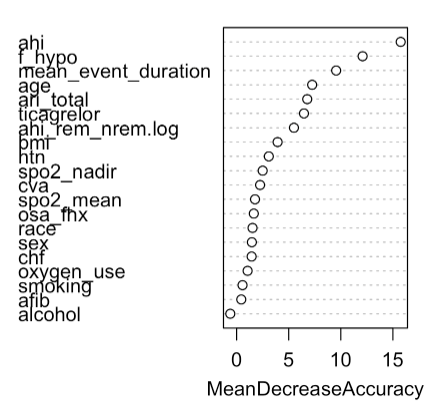


# E-Appendix 7. Model Development and Validation In the Subgroup of Patients with Moderate/Severe OSA

To explore if there may be better models to predict loop gain in patients with moderate/severe OSA we repeated the analyses described in the main manuscript in the subset of patients with an AHI>15/h (N=733 of the original 1055).

1. Predicting Continuous Loop Gain

The model with 2 predictors (Apnea-hypopnea index [AHI], percentage of hypopneas) had the lowest estimated test error (mean square error, MSE) based on 10-fold cross validation (red dot; red error bar = 1 standard error). However, the one standard error rule suggested similar performance for the 1-predictor model (AHI only; green dot):

Of note, the 2-predictor model included the same predictors as the 2-predictor model identified in the full cohort (Table 2 in the main manuscript) but the cross-validated mean-square error (MSE) was substantially worse (0.037 vs 0.0347; see E-Appendix 1), perhaps due to greater imprecision of coefficient estimates owed to the smaller sample size.

Alternative models showed similar performance as the 1-predictor linear regression model. Of note, the lasso model (using the 1 standard error rule) suggested a model including the same two predictors as the 2-predictor linear regression model with the smallest MSE (i.e., AHI and percentage of hypopneas), which were also among the top predictors of the random forest model again (details of the lasso and random forest models not shown).

The final 1-predictor linear regression model is shown in the table below.

|  | **Estimate** | **Standard Error** | **t value** | **p-value** |
| --- | --- | --- | --- | --- |
| (Intercept) | 0.562 | 0.0161 | 34.7 | <0.001 |
| Apnea Hypopnea Index (/h) | 0.00288 | 0.00030 | 9.62 | <0.001 |

When applying this 1-predictor model to the test set, there was no significant correlation between the predicted and the reference loop gain (r=0.09, P=0.25). Moreover, there was a suggestion of an overestimation bias for higher reference loop gains (i.e., the model tended to overestimate higher reference loop gains): as shown in the figure below, the 95% confidence interval of the least-squares mean of the reference loop gain (blue line) excludes/is below the line of identity for reference loop gains >~0.7.

2. Predicting Categorical Loop Gain (High vs Low)

Performance of the above 1-predictor (AHI) linear regression model for classifying subjects as high vs low based on the reference loop gain was very poor (AUC 0.53, 95%-CI 0.44 to 0.61). Logistic regression procedures performed on the subgroup of patients with moderate/severe OSA in the training set suggested a 4-predictor model (body mass index, AHI, percentage of hypopneas, event duration) to be superior (cross-validated AUC 0.71), but the performance in the test set was poor (0.59, 95% 0.50 to 0.67) suggesting overfitting.

3. Performance of the Original Model in the Subgroup of Patients with Moderate/Severe OSA

Of note, when applying the final 2-predictor model developed from the full cohort (see Table 2 in the main manuscript) to the subgroup of patients with moderate/severe OSA (i.e., AHI>15/h) in the test set, then performance was almost identical as when applied to all patients (i.e. AHI>5/h) in the test set (compared upper panels vs lower panels in the figure below).

Overall, these exploratory analyses suggest that the final model developed from the full cohort is also the optimal model for—and performs well in—the subgroup of patients with moderate/severe OSA.

# E-Figure 1. Comparison of Prediction Models of Continuous Loop Gain.


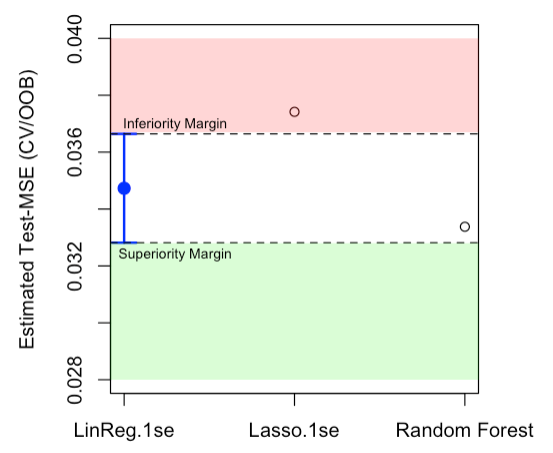


# E-Figure 2. Comparison of Prediction Models of Categorical Loop Gain (High vs Low). The estimated test AUC for the linear regression model based on 10-fold cross-validation was 0.674.


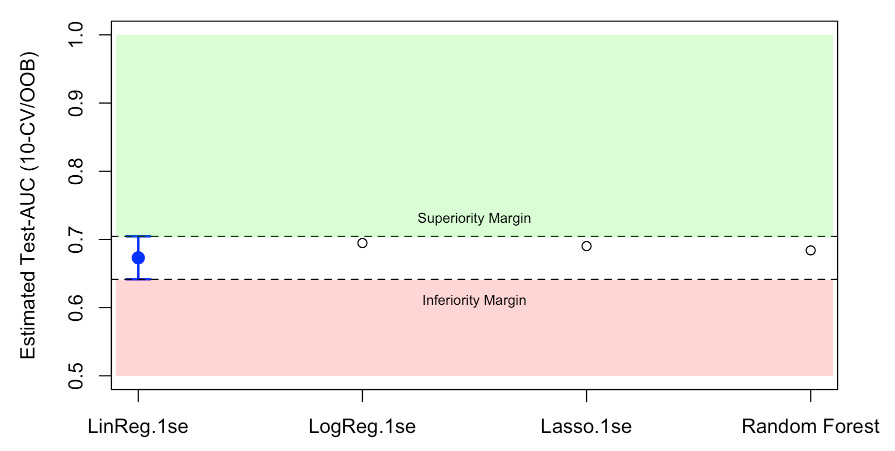

Supplement: Supplementary file 1 — Additional file 1. Online Supplement. [file 12890_2022_1950_MOESM1_ESM.docx]
